# 

**Published:** 1910-11

**Authors:** 


### TEXAS MEDICAL JOURNAL

*"Red Back"*

Established
July, 1885

PUBLISHED AT AUSTIN, TEXAS, BY

F. E. DANIEL, M. D., - - - - Proprietor.

PRINTED BY THE VON BOECKMANN-JONES CO.

**Subscription, $1.00 a Year, in Advance. - - - - Single Copies, 10 Cents**

Entered at the Postoffice at Austin, Texas, as second class mail matter.

